# Hybrid Pixel-Based Method for Cardiac Ultrasound Fusion Based on Integration of PCA and DWT

**DOI:** 10.1155/2015/486532

**Published:** 2015-05-18

**Authors:** Samaneh Mazaheri, Puteri Suhaiza Sulaiman, Rahmita Wirza, Mohd Zamrin Dimon, Fatimah Khalid, Rohollah Moosavi Tayebi

**Affiliations:** ^1^Faculty of Computer Science and Information Technology, Universiti Putra Malaysia (UPM), 43400 Serdang, Selangor, Malaysia; ^2^Cardiothoracic Unit, Surgical Cluster, Faculty of Medicine, 40450 Shah Alam, Selangor, Malaysia

## Abstract

Medical image fusion is the procedure of combining several images from one or multiple imaging modalities. In spite of numerous attempts in direction of automation ventricle segmentation and tracking in echocardiography, due to low quality images with missing anatomical details or speckle noises and restricted field of view, this problem is a challenging task. This paper presents a fusion method which particularly intends to increase the segment-ability of echocardiography features such as endocardial and improving the image contrast. In addition, it tries to expand the field of view, decreasing impact of noise and artifacts and enhancing the signal to noise ratio of the echo images. The proposed algorithm weights the image information regarding an integration feature between all the overlapping images, by using a combination of principal component analysis and discrete wavelet transform. For evaluation, a comparison has been done between results of some well-known techniques and the proposed method. Also, different metrics are implemented to evaluate the performance of proposed algorithm. It has been concluded that the presented pixel-based method based on the integration of PCA and DWT has the best result for the segment-ability of cardiac ultrasound images and better performance in all metrics.

## 1. Introduction

Image fusion is one of the major research fields in image processing. It is a procedure of combining the related info from two or several images, into a single image, without introducing any distortion which will be more informational and containing more details, and while it is more suitable for visual perception, it is complete in comparison with any of the inputs. Image fusion methods can enhance the quality and increase the application of input data [1]. For the purpose of the majority medical applications, medical image fusion aims to decrease ambiguity and minimize redundancy in result image when increasing the related info details [2, 3]. With the lately quick advances in the field of sensing technologies, multisensory systems have become a reality in medical imaging. As a result of using these technologies, we will have a huge increase in quantity of acquired data. Image fusion produces a useful way of decreasing that volume of information whilst, at the same time, extract all the valuable information from inputs. The goal of image fusion, except decreasing the quantity of info, is to build single enhanced image more appropriate for the purpose of human visual perception and for next image processing tasks like segmentation or feature detection in medical imaging.

Fusion also improves the capability for other applications by complementary information. In other words, the main condition for successful fusion is that “all” visible information in the input images should also appear visible in the fused image [4]. There are some needs of image fusion which are [5]

- extracting the whole desired information from the input images to get the relevant information,
- not introducing distortions or inconsistencies that will amuse human observers,
- robust and reliable to imperfections,
- improving reliability.

This paper is organized as follows: Section 2 provides a background and reviews image fusion concepts and some related works; Section 3 outlines proposed method for echocardiography fusion and explain the proposed algorithm; experimental results and evaluation of the proposed algorithm and discussion on results are presented in Section 4; the paper finishes with concluding remarks in Section 5.

## 2. Background and Related Works

### 2.1. Classification of Image Fusion Algorithms

Fusion is a procedure of incorporating the applicable info from a set of images of the same view right into a one image and the resulting image could be more useful and beneficial than any of the inputs [6]. The actual fusion process can take place at different levels of information representation.

**Figure 1:**
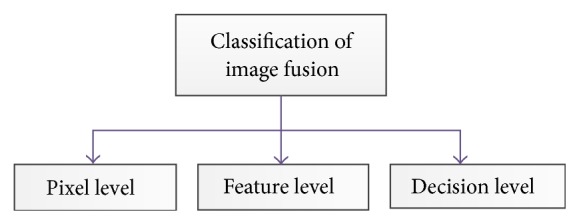
Classification of image fusion Algorithms.

Different categories of image fusion methods are usually classified in various levels: pixel, feature and decision level (see Figure 1).

1. Low-level or pixel-level: the pixel-level method works either in the transform domain or in spatial domain. They can directly work on the pixels of the images. Image fusion at pixel-level tries to incorporate low-level data, often in physical measurements like intensity [4] (see Figure 3).
2. Middle-level or feature-level: the feature level methods perform on characteristics taken out from the input images. They originally divide the image into contiguous areas and combine the areas together using their properties. The characteristics employed may be computed individually from every image or they may be acquired through the simultaneous procedure from all the images.
3. High-level or decision level: decision level fusion uses the results of initial object detection and classification as inputs to perform fusion as data integration [7].

**Figure 2:**
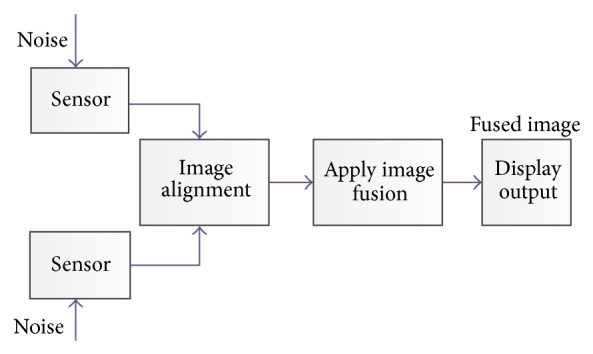
Pixel-level image fusion.

**Figure 3:**
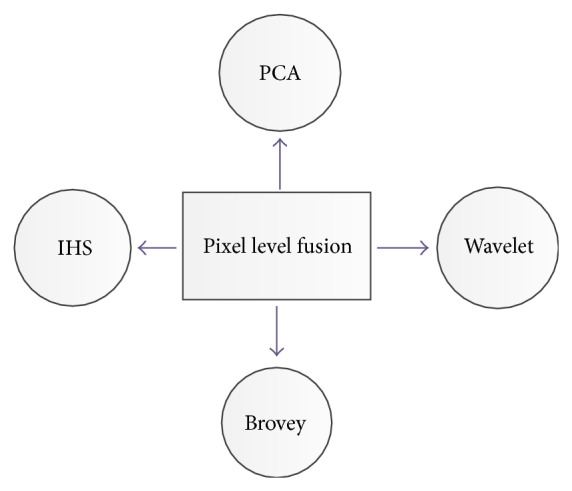
Some of well-known pixel-level image fusion techniques.

Pixel-level image fusion represents the visual information of the same scene from numbers of images which can be obtained using different sensors [4]. A simple diagram of a system using pixel-level fusion is demonstrated in Figure 2.

Details of some pixel-level techniques are described here [6].

**Figure 4:**
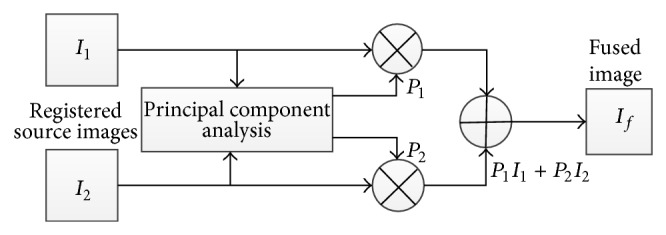
Flow diagram of information in first phase of proposed image fusion method.

(a) Simple maximum: in this technique, the combined image is acquired through choosing the maximum intensity of related pixels from two inputs:
  (1) $$\begin{array}{c} F\left(i,j\right)=\overset{m}{\underset{i=0}{\sum }}\;\overset{n}{\underset{j=0}{\sum }}\max A\left(i,j\right)B\left(i,j\right). \end{array}$$
 
*A*(*i*, *j*),   *B*(*i*, *j*) are inputs and *F*(*i*, *j*) is the resultant one.
(b) Simple mnimum: in simple minimum technique, the combined image is acquired through choosing the minimum intensity of related pixels from two inputs:
  (2) $$\begin{array}{c} F\left(i,j\right)=\overset{m}{\underset{i=0}{\sum }}\;\overset{n}{\underset{j=0}{\sum }}\min A\left(i,j\right)B\left(i,j\right). \end{array}$$
 
*A*(*i*, *j*),   *B*(*i*, *j*) are inputs and *F*(*i*, *j*) is the resultant one.

(c) Simple average: here, the combined image is acquired through calculating the mean intensity of related pixels from two inputs:
  (3) $$\begin{array}{c} F\left(i,j\right)=\frac{A\left(i,j\right)+B\left(i,j\right)}{2} \end{array}$$
 
*A*(*i*, *j*),   *B*(*i*, *j*) are inputs and *F*(*i*, *j*) is the resultant one.
(d) Weighted average: in weighted average technique, the combined image is acquired through calculating the weighted mean intensity of related pixels from two inputs:
  (4) $$\begin{array}{c} F\left(i,j\right)=\overset{m}{\underset{i=0}{\sum }}\;\overset{n}{\underset{j=0}{\sum }}WA\left(i,j\right)+\left(1-W\right)B\left(i,j\right) \end{array}$$
 
*A*(*i*, *j*), *B*(*i*, *j*) are inputs and *F*(*i*, *j*) is the resultant image and* W* is the weight component.
(e) Principal component analysis (PCA): PCA technique is a subspace one, which reduces the multidimensional data sets into lower dimensions for analysis. This method determines the weights for each source image using the eigenvector corresponding to the largest eigenvalue of the covariance matrix of each source image (see Figure 4).
(f) Discrete wavelet transform (DWT): these transforms have image decomposition tool that provide a variety of channels representing the image feature by different frequency subbands at multiscale. 2D discrete wavelet transformation (DWT) converts the image from the spatial domain to frequency domain (see Figure 6).
(g) Brovey transform (BT): Brovey transform also known as color normalized fusion is based on the chromaticity transform and the concept of intensity modulation. It is a simple method to merge data from different sensors, which can preserve the relative spectral contributions of each pixel but replace its overall brightness with the high spatial resolution image.
(h) Intensity hue saturation (IHS) method: the IHS method is a standard process in image fusion, with important restrictions in which only three bands are included. Basically, it was based on the RGB true color space. It provides the advantage that the separate channels outline certain color properties, namely, intensity (I), hue (H), and saturation (S). This special color space is usually selected because the visual cognitive system of human intends to treat these three elements as roughly orthogonal perceptual axes.

Finally, based on domains, image fusion methods are usually categorized into two groups.

- Spatial domain methods: with spatial domain approaches, we specifically deal with pixels of image. The pixel values are usually altered to acquire desired outcome.
- Transform domain methods: in transform domain methods, image is transferred into frequency domain first.

Fusion methods like averaging, Brovey method, principal component analysis (PCA), and IHS based techniques are categorized under spatial domain methods. The discrete wavelet transform is categorized under frequency domain approaches.

### 2.2. Echocardiography Image Fusion

Image fusion has turned into a popular term employed in medical diagnostics and treatment [8]. Fused images may be produced from a number of images from the similar imaging modality [9] or by merging details from multiple modalities [10] like MRI, CT, PET, and SPECT. For precise diagnoses, radiologists should incorporate information from multiple image formats. Fused, anatomically consistent images are specifically helpful in diagnosing and treating [11]. Some of applications of image fusion techniques in medical images would be fusing CT and MRI images, computer assisted surgery, and spatial registration of 3D surface [1].

Echocardiography imaging is a widespread technique to acquire cardiac images; however, it suffers from artifacts, high noises, and a limited field of view [12]. The created images are degraded by an implicit distortion named “speckle,” which comes from the destructive and constructive coherent summation of ultrasound echoes. The distortion caused by speckle can be defined as multiplicative noises that lead to granular look, degrade the contrast of the images, and decrease the ability to find information within the images. A technique to deal with these restrictions is using several images, selecting the best part from every image to provide a better quality result.

Some important requirements could be considered for fusion process: (a) the fusion process must keep the whole related info contained in the source images, (b) the fusion process should not produce any distortion or inconsistencies that would amuse the human observer or subsequent processing phases which can lead to a wrong diagnosis, and (c) irrelevant characteristics and noises need to be covered up to a maximum degree [13]. The problem that medical image fusion attempts to resolve is to fuse the information content from multiple images (or various imaging sensors) from the same view to achieve a fused image that contains the best possible details. Therefore, the fused image would produce improved superiority image compared to any of the original input images.

In this study, a pixel level based medical image fusion is introduced to display a fusion procedure creating a single fused image containing additional reliable information than individual input image. In spite of numerous attempts in direction of automation ventricle segmentation and tracking [14], the problem stay complicated study as a result of low quality characteristics of obtained images with missing anatomical details, or contains speckle noises, or restricted field of view. The simplest medical image fusion is to consider the mean of the gray level input images, pixel by pixel. However, using this technique on echocardiography images would produce several undesired effects and reduced feature contrast.

To overcome this problem, a new pixel based fusion method which integrates PCA and DWT is proposed in this paper. This fusion is achieved by weighted average of images to be fused. The weights for every input image are acquired from the eigenvector related to the largest eigenvalue of the covariance matrices of every input. Then, wavelet transform is used which presents directional details in decomposition levels and includes distinctive information at various resolutions. Performance metrics are used to evaluate the DWT, PCA, and the proposed algorithm performance as well [13].

## 3. The Proposed Method

A fusion approach which tries to provide a better quality image for improving the segment-ability of echocardiograms is proposed here. It is also capable of improving the contrast, decreasing the impact of echo artifacts, expanding the field of view, and improving the signal to noise ratio, as well. The proposed technique weights details of images, based on combinations of features of PCA incorporating with DWT between all the overlapping images to develop the fusion. The objective of this research is to implement spatial fusion on multiple echocardiography sequence and perform clinical examination in cooperation with cardiologists from UiTM, Sungai Bulah hospital, Malaysia.

The speckle should be filtered out without affecting salient characteristics in cardiac medical ultrasounds. Two main denoising approaches are employed: (a) fusion, which combines echocardiograms of the same area to provide a single decreased speckle image; (b) filtering.

This paper tries to optimize and continue the development of image fusion utilizing integration PCA with DWT, two well-known image fusion methods. PCA method can preserve a better resolution, but it distorts spectral features with various degrees as well. However, DWT will produce directional details in decomposition levels and includes special information at various resolutions. Additionally, DWT and PCA can keep more spectral details and spatial features, respectively. Also, PCA method can get salient characteristics to decrease redundancy. We present a spatial frequency (SF) motivated approach which integrates their advantages and improves image quality to avoid distortions and artifacts.

Lately, fusion echocardiography sequences are introduced through obtaining several echocardiography images with small changes in probe location and combining all of them together following alignment.

The particular idea of fusion echocardiography sequences allows us to enhance image quality as well as improving anatomical details which leads to better feature detection.

### 3.1. Principal Component Analysis

The PCA consists of a mathematic process which converts multiple correlated variables to several uncorrelated variables named principal components. Also as a linear transformation, it calculates a compressed and optimal explanation of dataset [13]. The 1st principal component accounts for the maximum amount of the variance in dataset as is achievable and every following component considers as the maximum amounts of the remaining variance as is available. First principal component is considered to be along the direction with the highest variance. The second principal component is restricted to put in the subspace perpendicular of the first one. Within this subspace, this component points the direction of maximum variance. The third principal component is considered in the maximum variance direction in the subspace perpendicular to the first two and so forth. PCA helps to minimize unnecessary information and emphasize the components with highest effect to improve signal to noise ratio metric. It is known as hoteling transform or the Karhunen-Loève transform as well [4].

### 3.2. Wavelet Transform

The PCA image fusion technique operates under spatial domain. However, the spatial domain fusion may produce spectral degradation. This is particularly crucial if the images are supposed to fuse such as echocardiography images were not acquired at the same time. Therefore, compared with the ideal result of the fusion, if this method is applied alone, it will produce poor and undesirable result for echocardiography images. Wavelet transforms are a decomposition tool for multiresolution images that offer a range of channels which represent the image characteristic through various frequency subbands. Since it has been found that wavelet fusion techniques outperform the standard fusion techniques in spectral quality, especially in minimizing distortion, we use both PCA and wavelet transform to get their advantages and minimize their disadvantages.

The proposed method that combines the PCA method with wavelet transform provides outstanding outcomes compared to standard PCA or wavelet transform alone. Wavelet transforms are generally classified into three categories; continuous, discrete, and multiresolution based [4]. In discrete wavelet transform, while decomposition is applied, the estimation and information element can be different. 2D DWT transforms echocardiography image from spatial domain to frequency one. Input is separated to horizontal and vertical outlines and shows the DWT first order; then image is divided to 4 areas which are LL1, LH1, HL1, and HH1. When decomposition is performed, the L-L band provides the typical image info while other bands include directional information caused by spatial orientation. Higher complete wavelet coefficients value within the high bands related to important characteristics include lines or edges. As a result, in wavelet transform, the size of image is halved in spatial direction at every decomposition level of procedure, therefore ending to a multiresolution signal representation. The main phase for combination is the creation of combination pyramid.

### 3.3. The Proposed Algorithm

The proposed algorithm consists of two phases. Suppose the inputs are sorted in 2 column vectors. In first phase, steps to project this data into 2D subspaces are as follows.

(1) At first, the data should be organized in column vector. Suppose* R* is the result column vector of dimension *n* × 2.
(2) After dividing the data into columns, the dimension of empirical average with each column is 1 × 2.
(3) Subtract average from every* R* column. The dimension of result matrix* X *is *n* × 2.
(4) Calculate covariance matrix* C* from matrix* X*.
(5) Take into account first column of eigenvector *V* that is related to larger eigenvalue *V*(1) to calculate normalized component *P*_1_ and *P*_2_ as
  (5) $$\begin{array}{c} P_{1}=\frac{V\left(1\right)}{\sum V},\;\;P_{2}=\frac{V\left(2\right)}{\sum V}. \end{array}$$

**Figure 5:**
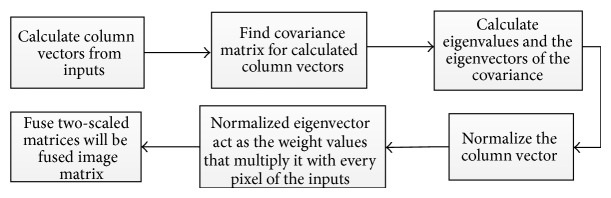
Stepwise procedure for first phase of proposed method.

In first phase, image fusion will be done by PCA. The information flowchart of the PCA algorithm is shown in Figure 5. The inputs *I*_1_(*x*, *y*) and *I*_2_(*x*, *y*) are ordered in 2 column vectors and their empirical averages are subtracted. The result vector has a dimension of *n* × 2, in which *n* is length of every image vector. Calculate eigenvector and eigenvalues for the result vector and eigenvectors related to bigger eigenvalue acquired. The normalized components *P*_1_ and *P*_2_  (i.e., *P*_1_ + *P*_2_ = 1) using 5 are calculated from the acquired eigenvector. The result image will be

(6) $$\begin{array}{c} I_{f}\left(x,y\right)=P_{1}I_{1}\left(x,y\right)+P_{2}I_{2}\left(x,y\right). \end{array}$$

There is not a restricted list of basic vectors such as wavelet, FFT, and DCT in PCA and it has its basic vectors rely on dataset. Suppose *X* is a random *D* dimensional vector which has zero empirical average. Orthonormal projection matrix *V* will be like *Y* = *V* ^*T*^ *X* with the subsequent restrictions. The *Y* covariance, that is, cov(*Y*), is a diagonal one and also it is inverse of *V* which is equal to its transpose (*V* ^−1^ = *V* ^*T*^). By using matrix algebra, we have

(7) covY=EYYT=EVTXVTXT=EVTXXTV=VTEXXTV=VTcovXV.

Multiplying each side of formula 7 by *V*, one gets

(8) $$\begin{array}{c} V\mathrm{cov}\left(Y\right)=VV^{T}\mathrm{cov}\left(X\right)V=\mathrm{cov}\left(X\right)V. \end{array}$$

By writing *V* as *V* = [*V*_1_, *V*_2_,…, *V*_*d*_] and cov(*Y*) as

(9) λ10…000λ2…00⋮⋮⋱⋮⋮00⋯λd−1000⋯0λd

substituting 7 into 8 gives

(10) λ1V1,λ2V2,…,λdVd   =covXV1,covXV2,…,covXVd.

It could be rewritten as

(11) $$\begin{array}{c} \lambda_{i}V_{i}=\mathrm{cov}\left(X\right)V_{i}, \end{array}$$

where *i* = 1, 2,…, *d* and *V*_*i*_ as an eigenvector of cov(*X*).

**Figure 6:**
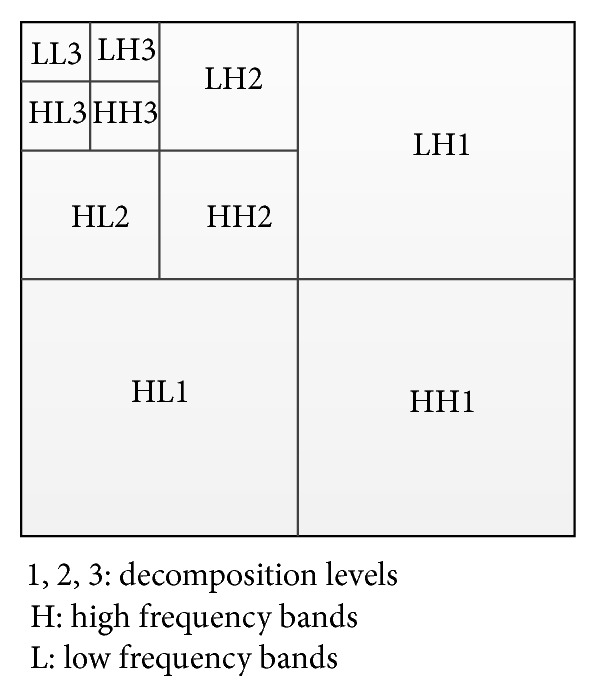
Wavelet decomposition in 3 levels.

In the second phase, in order to provide superior outcomes, we will apply wavelet fusion on images which were obtained from the first phase (see Figures 8 and 9). In this phase, while decomposition is applied, the estimation and information element can be different. 2D DWT changes the echocardiography images domain from spatial to frequency. First, the image is separated to horizontal and vertical outlines, and then it shows DWT first order, through the image which is divided to 4 areas that are LL1, LH1, HL1, and HH1 (see Figure 6). A certain signal of specific energy is projected on a continuous family of frequency bands (or identical subspaces of the function space *L* ^2^(*ℝ*)). For example, the signal might be displayed on each frequency band of the form [*f*, 2*f*] for all constructive frequencies *f* > 0. Next, the main signal can be rebuilt by an appropriate integration overall the producing frequency components [15–17]. The frequency bands or subbands are scaled versions of a subspace at scale 1.

This subspace consequently is in the majority circumstances created by the shifts of one generating function *ψL* ^2^(*R*), the mother wavelet. For instance, for scale one frequency band [1, 2] the function is given by

(12) $$\begin{array}{c} \Psi \left(t\right)=2\sin c\left(2t\right)-\sin c\left(t\right)=\sin\left(2t\right)-\frac{\sin\left(t\right)}{t}. \end{array}$$

Analyzing a signal utilizing all wavelet coefficients is extremely hard and computationally impossible, so maybe it is adequate to select a discrete subset of the higher half plane in order to construct a signal from the equivalent wavelet coefficients. For some actual variables *a* > 1, *b* > 0. The corresponding discrete subset of the half plane consists of all the points (*a* ^*m*^, *na* ^*m*^ *b*) with integers *m*, *n* ∈ *ℤ* ^2^. The equivalent baby wavelet is given by

(13) $$\begin{array}{c} \psi_{m,n}\left(t\right)=a^{-m/2}\psi \left(a^{-m}t-nb\right). \end{array}$$

An acceptable condition for construction of any signal *x* of specific energy through equation

(14) $$\begin{array}{c} x\left(t\right)=\;\underset{m\in Z}{\sum }\;\underset{n\in Z}{\sum }\left(x,\psi_{m,n}\right)\cdot \psi_{m,n}\left(t\right). \end{array}$$

The function {*ψ*_*m*,*n*_ : *m*, *n* ∈ *ℤ* ^2^} gives tight shape of *L* ^2^(*ℝ*).

**Figure 7:**
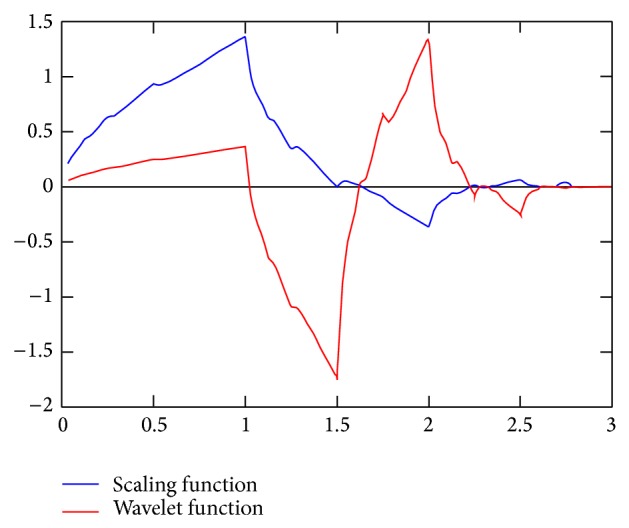
Daubechies 4 tap wavelet.

**Figure 8:**
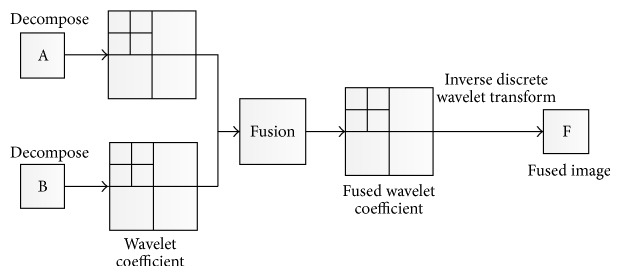
Second phase process for the proposed method.

**Figure 9:**
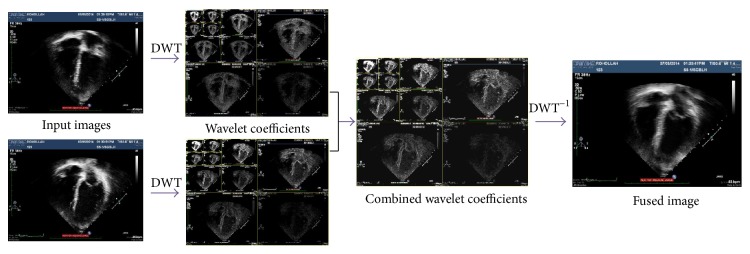
Applying second phase of proposed algorithm on two input images.

In a discrete wavelet transform, there are simply a limited number of wavelet coefficients for every bounded rectangular area in the top half plane. However, every coefficient needs the analysis of an integral. To prevent this mathematical complexity, an auxiliary function is needed, the father wavelet *ϕ* ∈ *L* ^2^(*ℝ*). Additionally, one has to limit *a* to be an integer. A typical selection is *a* = 2 and *b* = 1. The most popular two of father and mother wavelets is the Daubechies 4 tap wavelet (see Figure 7) [18–22]. We can construct the subspaces with the mother and father wavelets [15]:

(15) Vm=span⁡ϕm,n:n∈Zwhere  ϕm,nt=2−m/2ϕ2−mt−n,Wm=span⁡ψm,n:n∈Zwhere  ψm,nt=2−m/2ψ2−mt−n.

Let *S*(*n*_1_, *n*_2_) be input data and its size is *n*_1_ × *n*_2_; then the function of wavelet and scaling are

(16) w∅j0,k1,k2  =1N1N2∑n1=0N1−1 ∑n2=0N2−1sn1,n2∅j0,k1,k2n1,n2,wϵj0,k1,k2  =1N1N2∑n1=0N1−1 ∑n2=0N2−1sn1,n2∅j0,k1,k2n1,n2.

The algorithm of second phase for the proposed technique is as follows.

1. Implement discrete wavelet transform on image which is obtained from first phase to build lower wavelet decomposition.
2. Combine every decomposition level.
3. Hold inverse discrete wavelet transform on combined decomposed level, meaning rebuild the image, when the image rebuilt is F, the fused image.

## 4. Result and Discussion

**Figure 10:**
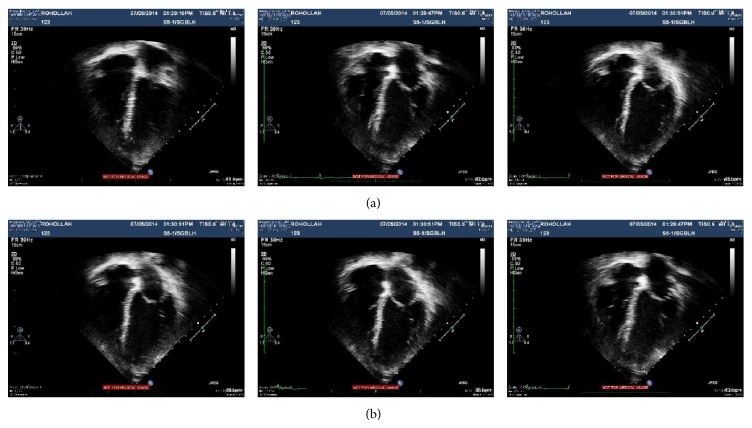
Original cardiac ultrasound images; (a) first row, dataset number 1, and (b) second row, dataset number 2.

In this section, it has been shown that the proposed approach can provide more satisfactory outcomes, compared to other techniques and algorithms in two aspects of visual effect and quantitative analysis. The evaluations are organized to employ well known image fusion approaches with four image quality measurements. Experiments have been done on the dataset which contains 10 subject cases. For every subject case, two echocardiographic phases have been considered which are different frames of heart cycle that have been obtained utilizing a Philips iE33 cardiac ultrasound scanning device (Philips Medical Devices, Sungai Bulah hospital, UiTM, Malaysia), under supervision of cardiac surgeon (see Figure 10). The presented integration of PCA and DWT fusion method is employed on the two mentioned phases of dataset. Also, PCA and DWT fusion techniques are employed on the data separately, and then the performance of every method is computed by applying four quantitative measurements. RMSE (root mean square error) is the appropriate measurement which shows how images are close together. Considering the possible artificial distortion over the combination procedure can also raise the entropy or spatial frequency measures of the combined image; the image quality index (IQI) is a reasonably trustworthy measurement for images with no reference image because IQI gets a value (in the range of 0 to 1) about how related is the result to both inputs.

### 4.1. Numerical Results

The typical requirement of a fusing procedure is to keep the whole useful info from the input images, whilst it should not produce any artifacts in resulting image. Efficiency measurements are employed to calculate the advantages of fusion and also are helpful to make comparison between results achieved with various techniques. Four measures are employed to examine image quality, including CC (correlation coefficient), IQI (image quality index), RMSE (root mean square error), and OCE (over-all cross entropy) [23–25]. The overall cross entropy is used to find the difference among two input images and the result one. Small measure is related to fine acquired result. The result of proposed integration of PCA and DWT method is compared with two results which are obtained by applying DWT and PCA alone.  Table 2 demonstrates the experimental result. From the measurements, it can be seen that the CC and IQI are the biggest with the presented technique. The RMSE and OCE of the proposed technique are least in 2 sets. It is indicated that the presented technique has the best results for fused images.

### 4.2. Experimental Results

**Figure 11:**
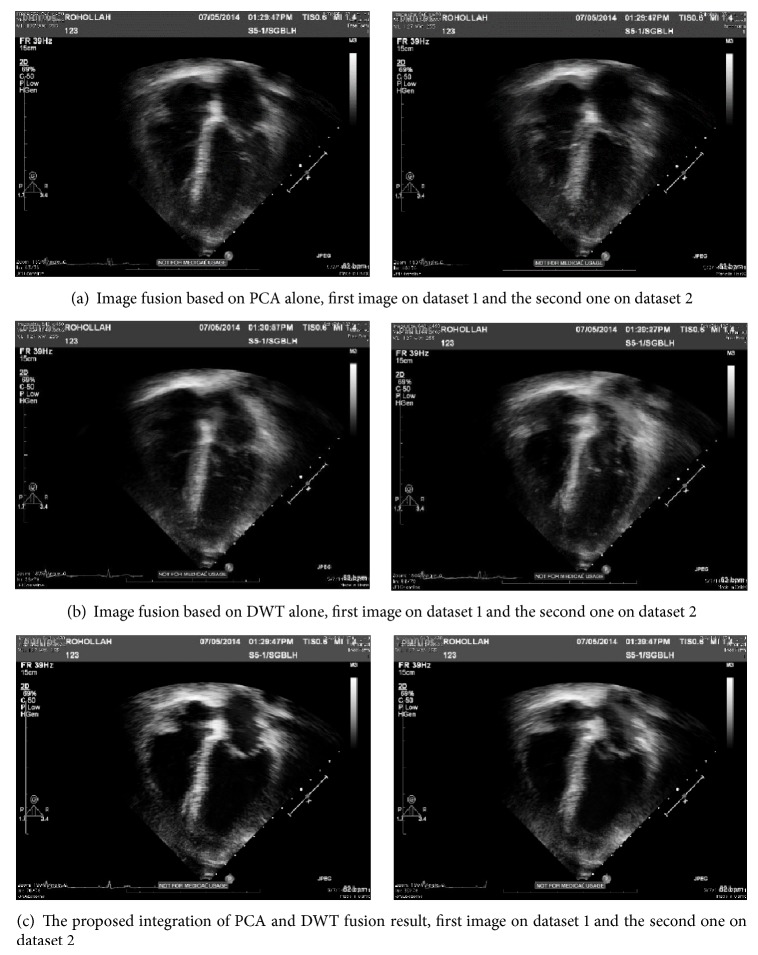
Results of applying PCA, DWT, and the proposed approach on dataset number one and dataset number two.

For evaluation, two types of comparison have been considered; first, result of proposed method is compared with result of other techniques, separately. Then, segmentation approach is used to evaluate the ventricle contours segment-ability on the fused image and input image both. To assess the proposed approach performance, two groups of echocardiograms are selected (as it is shown in Figure 11). All echocardiograms have the identical size of 1149 × 862 pixel.

#### 4.2.1. Evaluation by Result Comparison

The result of PCA method for the two mentioned datasets is shown in Figure 11(a). It can be simply viewed that image fusion based on PCA alone provides blurred information of tissues especially in heart ventricles. Image fusion based on DWT alone produces a result without clear boundary for ventricles and walls as well (see Figure 11(b)). As it can be seen, the best image fusion outcome is acquired through employing our proposed integration of PCA and DWT fusion method, as it is shown in Figure 11(c).

The characteristics and precise details displayed in result of proposed approach are significantly better compared to other fusion images. The image information such as tissues is improved clearly. Additional valuable information such as ventricle borders and shape is nearly completely achieved.

#### 4.2.2. Evaluation by Segment-Ability

This section provides experiments for the examination of ventricle border segment-ability on the fused image from three methods and input image as well. The ventricle segment-ability is particularly described in the following as the capability of the image for being effectively segmented utilizing a segmentation method. A level set segmentation method which is proposed in [26] is used to show the segmentation result on every image; so, comparison can be effectively done. This algorithm is a geometrically constrained level set segmentation which does not need a training or even prior shape approach and also uses intensity info within the particular image [26] (see Figures 13 and 14).

End systole and end diastole frames of every sequence were recognized by means of a specialist echocardiographer, based on the American Society of echocardiographic guideline [27]. Then the level set segmentation is applied on both phases and end systole and end diastole frames. First, the image was divided into four parts, and then the segmentation process began by putting some sort of elliptical shape of 10 pixels radius in the right bottom square or left bottom square. The variable values assigned for the right and left ventricles' segmentation approach are described in Table 3. The variables of feature detection, in other words edge indicator, as well as segmentation, were maintained identical between the fused image and input one, to examine the actual level of image quality sensitivity.

Effective convergence of the segmentation procedure, to achieve close to the left ventricle endocardial boundary, was quantified and the failing of the technique was categorized visually as a result of these probable reasons: (1) ventricle cavity speckle; (2) border loss because of inadequate border info; and (3) both loss and speckle.

In order to verify the measurements, the fused images are segmented manually by a specialist echocardiographer. Ventricles' endocardial boundaries are manually tracked in all planes of the end diastole as well as end systole. Left ventricle trabeculations with papillary muscles are integrated inside the left ventricle cavity, based on the American Society of echocardiographic guideline [27]. The actual result of left ventricle is regarded as source for assessment of segmented one, while employing the validation measurements will be explained in the following.

*Validation Methods*. The ventricles' endocardial acquired through geometrically constrained level set segmentation technique [26] is compared with contour extracted from manual delineation. The subsequent quantitative methods are applied to measure similarity among automated and source contours.

(1) DSI (dice similarity index) is calculated as a way of measuring the agreement among the contour (*V*) of automated technique and the source contour (*V*_ref_), providing a rating value between 0 and 1 (0: no agreement, 1: full agreement). DSI is calculated as
  (17) $$\begin{array}{c} \text{DSI}=\frac{2\left(V\cap V_{\text{ref}}\right)}{V+V_{\text{ref}}}, \end{array}$$
 in which ∩ stand for the intersections between the 2 contours.
(2) Mean surface distance: this measure is defined as *d*_mean_, among the surface (*S*) coming from automated technique and the source surface (*S*_ref_) described as
  (18) $$\begin{array}{c} d_{\text{mean}}=\frac{1}{2}\left[d\left(S,S_{\text{ref}}\right)+d\left(S_{\text{ref}},S\right)\right], \end{array}$$
 where *d*(*S*, *S*_ref_) is the average of distances between every surface pixel in *S* and the closest surface pixel in *S*_ref_, while *d*(*S*_ref_, *S*) is calculated in the same way.

**Figure 12:**
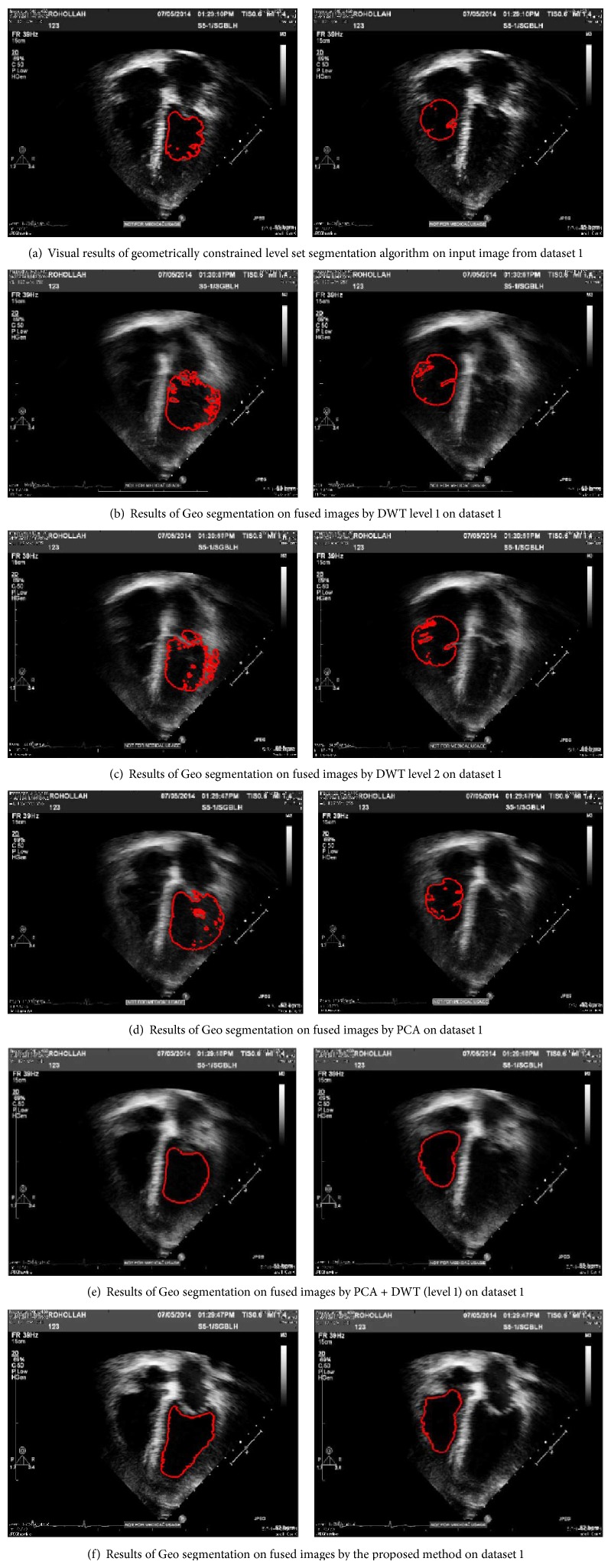
Visual results of segmentation on end-diastole frame for fused image and input image both; reference image segmentation results have some failure due to leakage.

**Figure 13:**
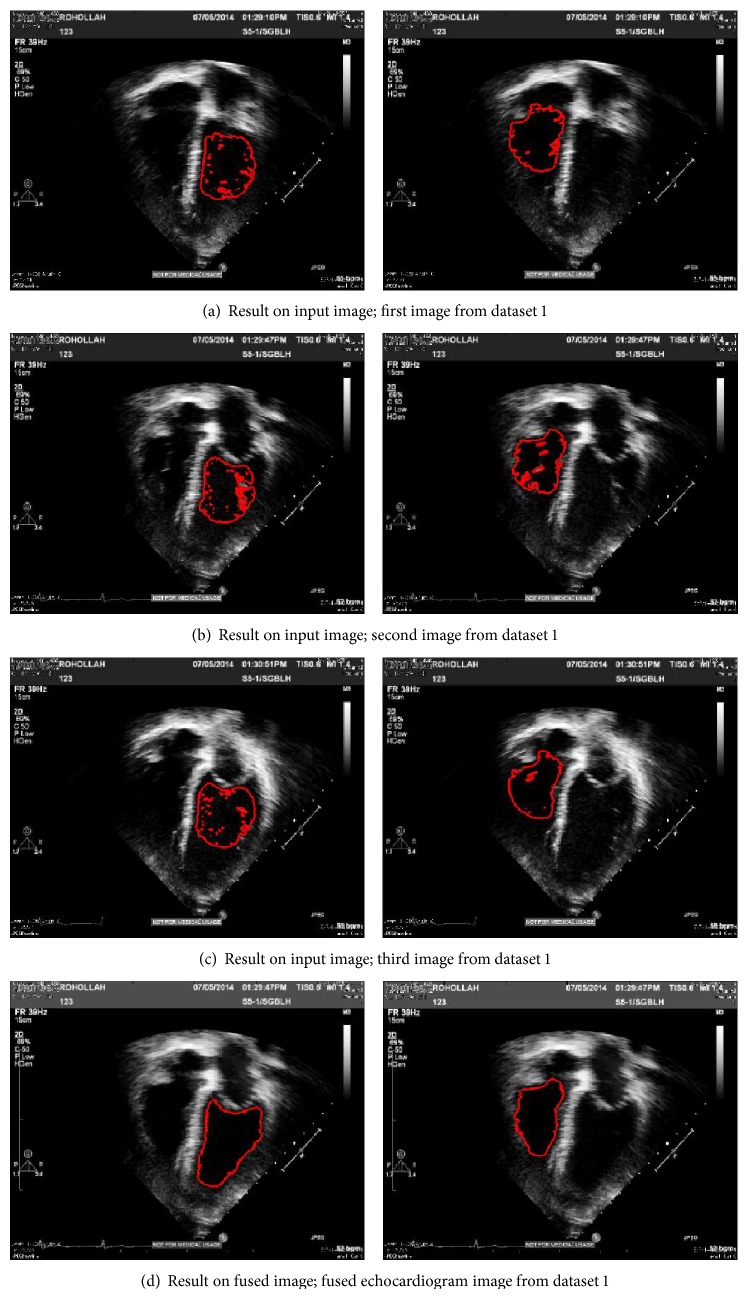
Results of segmentation of end systole frame; comparison between results on input images from dataset 1 by fused image from the same dataset.

**Figure 14:**
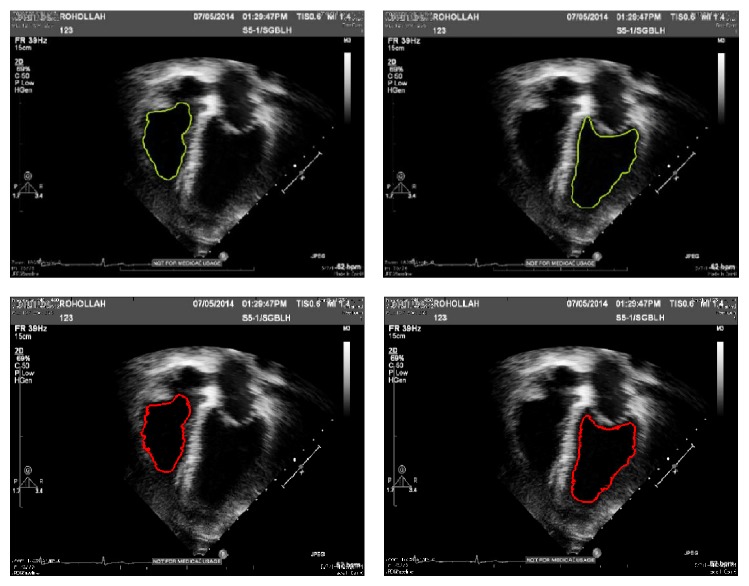
Comparison between manual delineation by expert and segmentation result from proposed method. Green contours: source segmentation through manual delineation done by expert. Red contours: acquired by employing geo segmentation technique on fused echo. The outputs indicate a near agreement among the automated technique and manual delineations.

Figure 12 presents the graphic segmentation results for two sets of subject cases visualized on plane of images from fused echocardiography and input image both. It demonstrates a case of effective left ventricle and right ventriclesegmentation at end diastole on the fused image, with a failing on the input image. In this case, the failing is a result of lacking enough image data in addition to cavity noise which in turn leads to loss of the endocardial surface expanding exterior of the correct border.

As it is shown, there is a good result for the fused image, because of lots of noise in left ventricle cavity and a smaller amount observable anatomical info in input image than the fused one.

The segmentation result which was explained above shows that the segmentation technique acts more superior on the fused echocardiograms than on input images. This can be, essentially, a direct effect of enhancement in perfection of echocardiogram anatomical description and also improvement in image quality because of multiple image fusions. The final result indicates that the fused images are superior fitted to ventricles' endocardial segmentation qualitatively and quantitatively (Table 1).

The particular amount of segmentation failures for every failing function is computed.  Table 4 summarizes the percent of segmentation failures for fused images and input echocardiograms. For input image, the technique is unable in accurate segmentation in many instances at end diastole (87.3%) and over half the time at end-systole (61.7%). For fused images, the segmentation method is unable in accurate segmentation 24.6% of times at end diastole whilst there was just one failing at end systole (3.1%). This means that fusion results in enhanced image quality which consequently leads to effective ventricles segmentation.

It is seen that input echocardiograms possess greater quantity of ventricles cavity noise in accordance with fused images, as Table 4 shows that ventricles cavity noise is a significant cause of failure (27.8% in end diastole and 59% in end systole) on input images in comparison with absolutely no failures on the fused ones. Lastly, input images are far more impacted by the fused loss along with noise elements (57.8% at end diastole and 39% at end systole) compared to just one such situation (14.7% at end diastole) on fused ones.

Table 5 presents the comparability among automated and manually delineated contours, at end systole and end diastole. The assessment was done on 8 end systole and 6 end diastole contours, based on the quantity of effective ventricle segmentation on fused echocardiograms, using the validation procedures explained before (Table 4).

**Figure 15:**
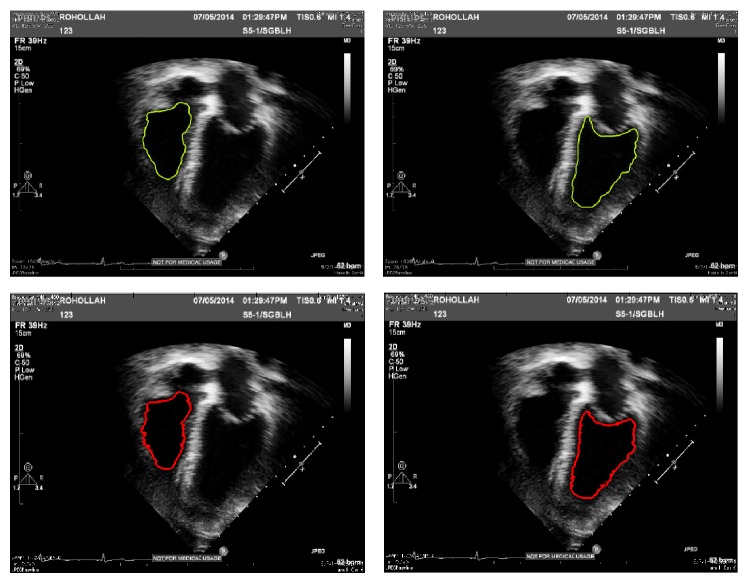
Comparison between manual delineation by expert and segmentation result from proposed method. Green contours: source segmentation through manual delineation done by expert. Red contours: acquired by employing geo segmentation technique on fused multiple view echo. The outputs indicate a near agreement among the automated technique and manual delineations.

Mean DSI measures of 0.91 and 0.79 in end diastole and end systole, respectively, indicate a superior overlap among the manually delineated and segmented contours. The distance failure among the automated and manual contour is smaller, pointed out through average distance of 2.26 mm and 1.64 mm at end diastole and end systole, respectively.  Figure 15 demonstrates cases with the automated and manual contours superimposed on the input echocardiograms in order to demonstrate the similarity among them.

### 4.3. Accuracy Evaluation of Endocardial Contour

Previously, the analysis has concentrated on image quality, info for segmentation of ventricles, improved repeatability, and achievement endocardial segmentation after fusion. Nevertheless, a crucial problem should be solved: will fusion displace the positioning of endocardials or maintain it in any case? We made a comparison of the endocardial exterior among the fused and input echocardiograms. A subset consists of 6 subjects (fused and input echocardiogram for every subject) and was chosen from database number 1. Those subjects are selected which demonstrated high quality on ventricle cover and anatomical description in single echocardiograms. The endocardial borders are delineated on fused and input echocardiograms both, by a well-experienced expert based on the standard protocol explained previously.

The extracted contours from delineation of fused image and input image are compared utilizing contour distance validation and DSI methods, explained before. The comparison results are presented in Table 6 showing a high agreement with 0.89 DSI and a modest distance failure about 0.95 mm.These outcomes demonstrate that there is high similarity among the endocardials delineated from fused image and input image. Also endocardials are maintained after fusion without obvious displacement in position.

### 4.4. Discussion

Evaluation has been performed utilizing volunteer and patient databases including ten series of echocardiography images. Result of the proposed method is compared with input (nonfused image) and with result of two other techniques. Experiments demonstrate that the proposed technique is able to get input images, degraded by artifacts, and provide a fused image with better quality. The visual evaluation is done by cardiologists and confirmed very good preference for the combined images regarding quality of image, expanded field of view, low cavity distortion, and high endocardial boundary description. Fusion echocardiographic images provide significant enhancement in anatomical details contained in the image and also in image quality. To analyze the result of this enhancement on automatic image analysis, this particular study described an organized process for evaluation the results using a geometrically constrained level set segmentation algorithm [26]. In this paper, the impact of increasing image info and quality in cardiac ultrasound images is explained objectively and quantitatively. The outcomes showed that fusion helps the automatic analysis in echocardiographic images, drastically.

## 5. Conclusion

In this study, a new fusion technique for echocardiography images has been presented based on integration of PCA and DWT. Experimental results indicated that the presented technique is effective in fusion echocardiography images and outperforms state-of-the-art developed approaches in quantitative and qualitative evaluation. Statistical and visual comparisons showed that the fusion result of the proposed method include more info, while artifacts are so small. In addition, the presented method can produce more acceptable outcomes, compared to other techniques in the two aspects of visual effect and quantitative analysis. Different metrics are employed to examine the performance of algorithm and it is shown that using discrete wavelet transform with higher level of decomposition incorporating with principal components analysis has better performance in all metrics.

This study mainly evaluated the quality as well as info of fused image and input image for segmentation. For segmentation, a geometrically constrained level set segmentation algorithm [26] was employed to show the result. This level set segmentation algorithm was employed on fused images and input images both. The qualitative and quantitative outcomes demonstrated that segmentation approach performed superiorly on the fused images more than input ones.

Further researches will try to examine ventricles' tracking and also evaluate the segment-ability of myocardial muscle which is more difficult than endocardial boundary. Furthermore, the motion approximation can be done for 3-dimensional strain evaluation, mainly because it basically offers a dense motions field. This study has shown the result of fusion echocardiography on automatic image segmentation for the specific echocardiographic images. The idea of fusion could be definitely placed on other fields of ultrasounds, for instance, fetal echocardiographic images [9]. Generally, we anticipate that the same developments of enhanced functionality in automatic analysis could be found in some other field of imaging of ultrasound. Therefore, fusion has a significant part to perform in ultrasound analysis to boost quantitative analysis.

## Figures and Tables

**Table 1:** Advantages and disadvantages of the various image fusion methods.

Method	Domain	Advantages	Disadvantages
PCA (principal component analysis)	Spatial	(i) Fused images have high spatial quality (ii) Extract main features to minimize redundancy (iii) Preserve more spectral information contents (iv) Keep a better resolution	(i) Artifacts of spectral feature images and the reference low resolution images with different degrees (ii) Spectral degradation (iii) Not suitable for medical images

DWT (discrete wavelet transform)	Transform	(i) Have least spectral distortion (ii) Preserve more spatial features (iii) Produce directional info in decomposition level and include salient information at various resolution (iv) Give a good signal to noise ratio compare to pixel based approaches	(i) Less spatial resolution for final fused image (ii) Not suitable for medical images

Proposed method (integration of PCA and DWT)	Transform	(i) Integrate PCA and DWT advantages and powerfully improve fused image quality to avoid distortions (ii) Preserve more spatial features and more spectral information contents (iii) Extract main features to minimize redundancy (iv) Output image will contain high spatial resolution and also high quality spectral content	—

**Table 2:** Comparison of image fusion techniques result; IQI is image quality index; CC is correlation coefficient; RMSE is root mean square error; and OCE is overall cross entropy.

Input	Method	IQI	RMSE	CC	OCE
Dataset 1	**Proposed**	**0.1987**	**0.1041**	**0.9872**	**0.5987**
	PCA	0.1623	0.1252	0.9829	0.9170
	DWT	0.1527	0.1289	0.9819	0.8701

Dataset 2	**Proposed**	**0.2135**	**0.0926**	**0.9798**	**0.4528**
	PCA	0.1678	0.1268	09662	0.5821
	DWT	0.1542	0.1287	0.9654	0.6012

**Table 3:** Experimental variables of ventricles' segmentation technique.

Border indicator				Level set			Postprocessing procedures	
Gaussian smoothing standard deviation	*σ*		1.6 or 2^a^	Balloon force weight	*α*	1	radius of circle, Apex point selection	3

Edge contrast parameter	*ν*		0.06 or 0.1^a^	Regularization weight	*β*	0.64	Surface smoothness (Gaussian), standard deviation	0.3

Edge exponent parameter	*λ*		2	Advection weight	*γ*	3	—	

^a^Because of dissimilarities in quality throughout the database, a small subset of echocardiograms was prepared with unique variables intended for smoothing and also border indicator calculation. Typically, this is performed as a result of varying image quality in input echocardiography and less often for fused ones.

**Table 4:** Failure of segmentation technique on fused and input echocardiograms and its particular quantification. The outcomes are demonstrated for 10 subjects. As explained before, segmentation failing means the inability of the segmentation algorithm to achieve the actual endocardial border caused by border loss, noise of cavity, or both equally.

	End diastole		End systole	
	Input Image	Fused image	Input Image	Fused image
Total failures	87.3%	24.6%	61.7%	3.1%
Cavity noise	27.8%	0%	59%	0%
Leakage + noise	57.8%	14.7%	39%	0%

**Table 5:** Failures for evaluation among surfaces from geometrically constrained image segmentation method and manual delineation at end diastole and end systole. DSI (dice similarity index) indicates that the quantity of agreement among the traced and source volumes. *d*_mean_ (surface distance mean) displays the standard distance, among the traced and source surfaces.

	DSI (mean ± std)	*d*_mean_ (mean ± std)
End diastole	0.91 ± 0.07	2.26 ± 0.78
End systole	0.79 ± 0.05	1.64 ± 0.45

**Table 6:** Comparison and evaluation of contours acquired from manual delineation of fused image and input image. The outcomes present a high similarity among them; it means endocardial contours stay stable after fusion and do not displaced through the combination procedure.

	Input image versus fused image
DSI [mean ± std]	0.89 ± 0.03
*d*_mean_ [mean ± std]	0.95 ± 0.34
